# Pilot-Scale Vinification of Cabernet Sauvignon Using Combined *Lactiplantibacillus plantarum* and *Saccharomyces cerevisiae* to Achieve Wine Acidification

**DOI:** 10.3390/foods11162511

**Published:** 2022-08-19

**Authors:** Jiao Jiang, Wenjing Zhang, Yitian Wu, Xuerong Shi, Xiaobing Yang, Yuyang Song, Yi Qin, Dongqing Ye, Yanlin Liu

**Affiliations:** 1 College of Enology, Northwest A&F University, Yangling, Xianyang 712100, China; 2 Ningxia Helan Mountain’s East Foothill Wine Experiment and Demonstration Station of Northwest A&F University, Yongning, Yinchuan 750104, China; 3 Shaanxi Engineering Research Center for Viti-Viniculture, Yangling, Xianyang 712100, China; 4 Guangxi Key Laboratory of Fruits and Vegetables Storage-Processing Technology, Guangxi Academy of Agricultural Sciences, Nanning 530007, China

**Keywords:** *Lactiplantibacillus plantarum*, *Saccharomyces cerevisiae*, wine acidity, lactic acid, volatile compounds, sensory analysis

## Abstract

Insufficient acidity in grape berries from warm climate regions has been exacerbated due to global warming, thereby becoming a major concern for winemaking. The wine lactic acid bacterium *Lactiplantibacillus plantarum* has potential to ameliorate wine acidity by producing lactic acid from hexose metabolism, but its impact on wine compositions and sensory outcomes is not well studied. Here, we evaluated acidification and fermentation performance of indigenous *L. plantarum* in two inoculation regimes (i.e., reverse inoculation and co-inoculation) by conducting pilot-scale vinification using Cabernet Sauvignon with low acidity. Important parameters of the bio-acidified wines, including fermentation kinetics, basic oenological parameters, volatile and sensory profile were compared to those in wines produced by single *Saccharomyces cerevisiae* with/without chemical acidification. Total titratable acidity in *L. plantarum* wines were either comparable or significantly higher compared to the chemical acidification control. Chemical profiling reviewed remarkable differences in certain organic acids and major volatile compounds, especially an up to a five-fold, six-fold, and nine-fold increase in lactic acid, ethyl lactate and isoamyl lactate, respectively. Changes in chemical compositions of the bio-acidified wines resulted in differentiated sensory perception compared to the control wines. Except having higher scores for “wine acidity”, the flavour profile of the bio-acidified wines was shifted towards “jammy fruit” and “butter” aromas. Together, these findings highlighted the applicability of using *L. plantarum* to induce biological acidification along with modulation of wine flavour.

## 1. Introduction

Winemakers have long been seeking riper fruits for the aim of producing fuller-bodied wines that are preferred by both the consumers and wine experts [1]. This trend has been exacerbated over the past two decades [2]. However, due to climate warming and extreme weather events, excessive sugar accumulation and loss of organic acids in grape berries upon ripening is frequently seen in warm regions and has become one of the major concerns for winemakers [3]. Such grapes not only exert difficulties for successful and timely completion of alcoholic fermentation, but also would easily induce inferior sensory properties often linked with unbalanced low acidity. Inadequate acidity may also increase the likelihood of microbial spoilage, which would be detrimental for both the chemical and sensory profile of the wine [4]. Thus, there is a strong demand to develop appropriate strategies to managing wine acidity.

Currently, insufficient wine acidity in warmer regions is commonly corrected via supplement of authorised organic acids such as tartaric, lactic and malic acids [5]. Alternatively, winemakers can opt for physical acidification of wines based on ion exchange [6], electrodialysis [5], and blending with wines made from earlier harvested grapes [7]. Albeit efficient, these approaches can be limited by extra costs resulting from additional requirements of labours and equipment, or by consumer perception. For example, wines made from grapes at an early ripening stage contributed to fresh green and red fruit characters rather than the jammy fruit aroma and palate that are favoured by many consumers [8]. In comparison, the application of acidifying wine microbes could offer an inexpensive and straightforward solution, with potential to modulate wine flavour.

Generally, acidifying wine microbes are capable of synthesizing considerable amounts of lactic acid that are more microbially and chemically stable with rounder flavour and mouthfeel. In recent years, growing interest has focused on using *Lachancea thermotolerans*, a non-conventional yeast, to enhance the overall quality of wines that suffer from insufficient acidity [9,10]. The yield of lactic acid by *L. thermotolerans* yeasts would lead to the decrease in wine pH by 0.01—0.5 units [9]. Varied pH changes often linked with differential lactic acid production capability by the yeast, which can be influenced by many factors, encompassing strains [9,10], inoculation regimes [9], and fermentation conditions (e.g., fermentation temperature and SO_2_ addition [10]). The most productive *L. thermotolerans* strain formed 16.6 g/L lactic acid under oenological conditions during fermentation [11]. Aside from *L. thermotolerans*, wine lactic acid bacterium *Lactiplantibacillus plantarum* (formerly known as *Lactobacillus plantarum* [12]) also has such potential [13,14].

Normally, *L. plantarum* (henceforth LP) can present on grape skins and at different stages during winemaking [15]. They are rod-shaped bacteria and are best known as an ideal alternative to *Oenococcus oeni* for efficient malolactic fermentation (MLF) in wines with high pH [16]. Apart from MLF, LP displays a homofermentative character for hexose, which allows this specific bacterium to generate lactic acid as the sole metabolite through hexose catabolism, known as lactic fermentation [13,17,18]. This special feature makes LP a promising candidate as an acidifying starter culture without the risk of producing acetic acid, which is the major component of wine volatile acidity. Onetto and Bordeu reported the inoculation of commercial LP 10 days prior to addition of *Saccharomyces cerevisiae* (henceforth SC) led to the production of up to 8.3 g/L lactic acid in Carménère wine, and a reduction of 0.4 units in pH [14]. In this scenario, lower acetic acid yield (0.40 g/L) was observed, whilst 0.65 g/L acetic acid was detected in wines produced with co-fermentations of *O. oeni* and SC [14]. In another study undertaken by Lucio and the colleagues, pure LP cultures generated lactic acid at concentrations ranging from 17.2 to 18.8 g/L, but information on acetic acid production was not provided [13]. The same study further showed that only a small proportion of sugar was utilised by pure LP fermentation, leaving 170 g/L and more residual sugar in the must [13]. The above findings indicate that depletion of sugar relies on the involvement of SC, which could be either inoculated shortly after the addition of LP (reverse inoculation) or simultaneously inoculated (co-inoculation) into the grape must.

Varied inoculation regimes may affect fermentation and lactic acid yield, potentially due to the antagonistic activities of yeast against bacteria through direct cell-to-cell contact or producing compounds that inactivate bacterial growth [19]. Among the inhibitory yeast metabolites, ethanol and medium chain fatty acids perturb cell membrane structure, resulting in leakage of intracellular materials and ultimately cell death [19]. In addition, increased acidity resulting from lactic acid production can also exert an inhibitory effect on bacterial growth via impairing hexose metabolism and ATPase activities [20]. Thus, monitoring LP population to ensure efficient lactic fermentation becomes crucial when utilising this tool to achieve biological acidification.

Beyond bio-acidification, LP also plays an important role in the development of wine aroma and flavour. LP possesses a wider array of enzymes that can retain functional under typical oenological conditions compared to the commonly used *O. oeni* in the current winemaking practice [16,17]. Simultaneous alcoholic and MLF undertaken by co-cultures of LP and SC induced multiple effects on wine composition. Concentrations of major volatiles, including ethyl and acetate esters, fatty acids and higher alcohols can show an increase, decrease or no change compared to the SC monoculture wines [21,22,23,24,25]. Accordingly, compositional modulations of flavour substances produced through certain metabolic activities of LP may alter sensory perception of the wines [21,22,23,25].

Taken together, both the reverse inoculation and co-inoculation of LP and SC strategies have the potential to induce biological acidification along with modifying wine flavour. Currently, multiple attempts have been made to employ simultaneous inoculation of LP and SC for more efficient and reliable MLF [21,22,23,25,26]. Nonetheless, these studies focused more on the impact of co-inoculation of SC and LP upon malic acid degradation rather than lactic fermentation. So far, very limited knowledge is available on the applicability of LP to acidify wines beyond its success in lab-scale fermentation trials, including its influence on fermentation, wine composition and flavour. To address these issues, herein we assessed the acidification kinetics and fermentation performance of mixed starter cultures composed of an indigenous LP strain and a commercial SC strain by performing pilot-scale Cabernet Sauvignon vinification. Wines obtained from co-culture fermentations were further assessed for their chemical compositions and sensory outcomes by comparing those parameters to yeast monoculture fermentation with/without chemical acidification. To the best of our knowledge, this study described for the first time on the performance of indigenous LP in inducing biological acidification at pilot-scale red wine production.

## 2. Materials and Methods

### 2.1. Pilot-Scale Vinification

Cabernet Sauvignon grapes with 250 g/L sugar, 5.1 g/L titratable acidity (as tartaric acid) and pH 3.70 were handpicked from the vineyard of Yuma Wine Co., Ltd. (Qingtong Xia, China) during the 2018 vintage. Handpicked grapes were immediately destemmed and crushed prior to being randomly aliquoted (75 kg) to 100 L stainless steel vessels. A total amount of 40 mg/L SO_2_ was supplemented at crush using potassium metabisulfite (80 mg/L) to prevent the growth of undesirable microbes. Four batches of fermentation were then set up in triplicate with inoculation modalities outlined below (see Section 2.3 Fermentation modalities). The cap was plunged every 24 h with simultaneous determination of residual sugar, titratable acidity, yeast and bacterial population until fermented to dryness (residual sugar <4 g/L). Fermentation was performed at 25 °C, and after alcoholic fermentation terminated, the wines were immediately pressed off using a basket press into 50 L glass containers. The resultant wines were then dosed with 50 mg/L SO_2_, and stored at 0 °C prior to subsequent chemical and sensory profiling.

### 2.2. Microbial Strains, Culture Conditions and Inoculum Preparation

The LP strain used in this study was isolated from an uninoculated wine fermentation (Northwest A&F University, Xianyang, Shaanxi, China 2017), following by molecular identification via 16S rDNA sequencing as outlined by Paz et al. [27]. Sufficient LP inoculum for the pilot-scale fermentation was prepared through a 4-step method. Briefly, the cryogenically preserved LP culture (−80 °C in 25% glycerol) was initially pre-cultured in 10 mL MRS broth, then incubated for 48 h, at 28 °C, until OD_600_ reached 0.8 and above. This 10 mL pre-cultured LP was transferred into 90 mL MRS and was harvested after 48 h incubation at 28 °C, followed by being sub-cultured again into 900 mL MRS broth in a Schott bottle, at the same temperature. Cells were harvested by centrifugation (2000× *g*), washed with buffered phosphate saline, re-suspended in 2 L filter sterilised (0.22 μm) Cabernet Sauvignon grape juice diluted by a ratio of 1:2 with ultrapure deionised water, and incubated at 22 °C until OD_600_ reached 0.8. Final inoculation of the triplicate pilot-scale fermentation was performed at a rate of 2% *v*/*v*, which was approximately 2 × 10^7^ CFU/mL.

A commercial wine yeast CECA (*S. cerevisiae*, Angel Yeast, Yichang, China) was used for co-fermentation with LP at 200 mg/L to yield an inoculum at approximately 5 × 10^6^ cells/mL. Rehydration and inoculation of the wine yeast was performed according to the manufacturer’s instructions.

### 2.3. Fermentation Modalities

Four batches of fermentation with different inoculation modalities/treatments were carried out. Batch A used the reverse inoculation strategy (henceforth PreAF-LP), where starter cultures of LP were inoculated in the grape must and grown 24 h prior to SC addition. Batch B employed a co-inoculation strategy, which involved simultaneous inoculation of LP and SC into the Cabernet Sauvignon must (henceforth S-LP). Batches C and D were both inoculated with SC monocultures but were different in grape must acidity adjustment. In Batch C, the must was chemically acidified with 1 g/L tartaric acid prior to the inoculation of SC (henceforth TA-SC), whilst in Batch D, the grape must was directly inoculated with the SC monoculture without any intervention on must acidity (henceforth D-SC).

### 2.4. Yeast and Bacterial Enumeration

Viable yeast and bacterial cell numbers were determined by serially diluting the samples by 1:10 with ultrapure deionised water followed by spreading 100 μL droplets of the diluted samples on YPD and MRS agar medium, respectively. In order to obtain more accurate bacterial enumeration, the MRS agar medium was prepared with supplementation of 10 mg/L cycloheximide (Sigma-Aldrich, Saint Louis, MO, USA) to prevent yeast growth prior to using. The plates were incubated at 28 °C for 2~4 days before the colonies were counted.

### 2.5. Profiling of Wine Composition

Basic oenological parameters of the resultant wine samples, including residual sugar, titratable acidity, volatile acidity and ethanol, were determined following the protocols reported in OIV-INT-00-2020 (OIV, 2020). The pH of the wines was measured using a pH meter (Sartorius PB-10, Göttingen, Germany). Malic, lactic, citric, tartaric and acetic acids were quantified by HPLC (Agilent 1100, Agilent Technologies, Santa Clara, CA, USA) with an HPX-87H Column (300 mm × 7.8 mm, BioRad, Hercules, CA, USA) under the following condition: mobile phase, 2.5 mM H_2_SO_4_; flow rate, 0.5 mL/min; column temperature, 60 °C; injection volume, 20 μL. Signals were detected using an Agilent G1315B diode array detector at 210 nm. The organic acids were quantified from external calibration curves of the corresponding standard solutions using Agilent ChemStation Software (Version B.01.03).

Volatile compounds were extracted and analysed using headspace solid-phase microextraction–gas chromatography with mass spectrometry (HS-SPME-GC-MS) as described by Lan et al. [28] with some modifications. Briefly, each wine sample (5.0 mL) was transferred to a 20 mL screw-cap glass vial containing 1.0 g NaCl and 10 μL internal standards (4-methyl-2-pentanol, 20 mg/L). The vials were incubated at 40 °C with 400 rpm agitation in a CTC CombiPAL auto sampler (CTC analytic, Zwingen, Swiss) for 30 min. Volatile extraction was performed subsequently using a 50/30 μm DVB/CAR/PDMS SPME fibre (Supelco, Bellefonte, PA, USA) for 30 min with continuous heating and agitation at 250 rpm and 40 °C. The fibre was then desorbed at 250 °C in the GC injector for 8 min using splitless inlet injection mode. Volatiles were subjected to gas chromatography–mass spectrometry analysis using an Agilent 6890 GC system combined with an Agilent 5975 MS detector. The GC-MS unit was equipped with an HP-INNOWAX capillary column (60 m × 0.25 mm × 0.25 μm, J&W Scientific, Santa Clara, CA, USA) for volatile separation. Volatile compounds were carried by ultra-pure helium with a flow rate at 1 mL/min. GC program started at 50 °C and held for 1 min, then ramped to 220 °C at a rate of 3 °C/min, and held at this temperature for 5 min. Temperature of both the transfer line and the ion source was set at 230 °C, while the quadrupole was maintained at 150 °C. Ion–electron impact spectra at 70 eV were collected in the range m/z 29–350 with scan mode at 0.2 s interval.

Standard calibration curves were obtained using volatile compound standards in a chemically defined medium containing 14% *v*/*v* ethanol and 5 g/L tartaric acid, at pH 3.8. Mixture of standard volatiles was then blended with 10 μL internal standard (4-methyl-2-pentanol, 20 mg/L), and analysed following the same HS-SPME-GC-MS protocol as outlined above. Volatile compounds were identified using the retention time of the corresponding volatile standards and the NIST library, and were quantified using five-point standard calibration curves [28].

Odour activity values (OAVs) of each identified volatile compound was used to indicate their contribution to wine aroma perception, and was calculated as follows [29]:

OAV = VC/OTD

where VC refers to the concentration of each individual volatile compound (μg/L), and OTD is the odour threshold of the corresponding volatile reported in wine/wine-like medium [30,31,32,33,34,35,36,37,38,39].

### 2.6. Sensory Analysis

A panel of 12 tasters, comprising 6 males and 6 females (aged between 21 and 30 years) from the College of Enology, Northwest A&F University (China) was recruited for the sensory study. All panellists were well educated in wine science and had extensive wine-tasting experience. The sensory panellists were first trained using a 54-aroma kit (Le Nez du Vin, France) for at least four weeks until their aroma perception accuracy reached 95% and above. A proportion of the Cabernet Sauvignon wines was used in the subsequent training session to familiarise the panellists with the tasting environment, wine samples and sensory evaluation procedure [30]. This was followed by formal sensory analysis where the panellists were asked to score the sensory attributes and overall quality of wine samples using a ten-point intensity scale (0–9). On the ten-point scale, 0 represented “absence of perception”, whilst 5 represented “moderate perception” and 9 was “the maximum perception”. Wine samples (30 mL) were equilibrated to room temperature prior to being served in odourless International Organisation for Standardisation (ISO) clear wine glasses covered with glass Petri dishes in random order.

### 2.7. Data Analysis

Raw data was first processed with Microsoft Excel 2016 (Microsoft, Richmond, VA, USA), and were expressed as mean values with standard deviation. Sugar consumption, yeast and bacterial population dynamics, and acidification kinetics were plotted with GraphPad Prism 9.0 (GraphPad Software, San Diego, CA, USA). The same software was also used to perform statistical analysis, including Students’ *t*-test to compare bacterial growth between the two LP inoculation modalities, and one-way ANOVA coupled with Tukey’s honest significant different (HSD) post hoc tests to compare the remaining measured parameters among four fermentation batches. A confidence interval for the Tukey’s HSD post hoc tests was set at 95%. The significantly different oenological parameters and volatiles were further subjected to principal component analysis (PCA) using XLSTAT (Addinsoft SARL, Paris, France).

## 3. Results and Discussion

### 3.1. Fermentation Kinetics and Trend of Titratable Acidity during the Pilot-Scale Vinification

**Figure 1:**
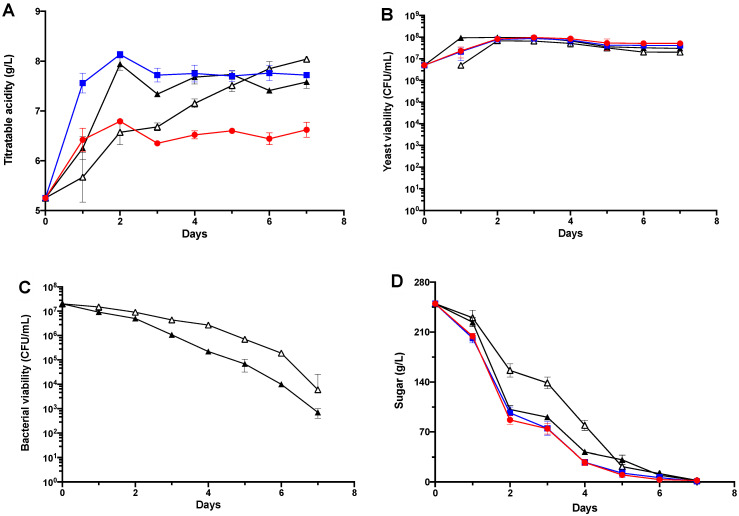
Fermentation and acidification kinetics in the Cabernet Sauvignon pilot-scale vinification. (**A**) Sugar consumption kinetics; (**B**) Yeast viability; (**C**) LP population; (**D**) Trends in total titratable acidity during fermentation. D-SC (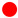), TA-SC (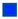), S-LP (▲), PreAF-LP (**△**).

Pilot-scale vinification was undertaken using Cabernet Sauvignon must with LP in two inoculation modalities with SC (PreAF-LP and S-LP). Titratable acidity (TA, expressed as tartaric acid) of the grape must was monitored and recorded daily during fermentation to assess the ability of LP to induce biological acidification (Figure 1A). The trends in TA for D-SC, TA-SC and S-LP showed initial increases at the onset of fermentation, followed by a slight decrease from Day 2, then fluctuated slightly from Day 3 till Day 7, with average TA around 6.5, 7.7 and 7.6 g/L, respectively. Acidification kinetics of the bio-acidified S-LP wines were comparable to the chemically acidified TA-SC wine control (*p* = 0.2885, Tukey’s HSD post hoc analysis), but were significantly more pronounced compared to D-SC (*p* < 0.05, Tukey’s HSD post hoc analysis). Interestingly, TA of PreAF-LP increased steadily throughout fermentation, finally reaching 8.04 g/L by the time alcoholic fermentation completed, which was the highest amongst all treatments. Similarly, Onetto and Bordeu [14] observed a continuous decrease in pH from 3.9 (grape must) to 3.4 (resultant wine) over the course of fermentation reversely inoculated with LP. Together, these findings highlighted the effectiveness of using LP to correct the inadequate wine acidity in warm wine regions.

Trends in yeast population during fermentation showed initial increase after inoculation, followed by reaching stationary phase from Day 2 onwards (Figure 1B). Tukey’s multiple comparisons test further indicated that the trends in viable yeast cell numbers were not significantly different between any two of the four batches (*p* > 0.05 in each situation). In terms of LP growth, viability drastically decreased for both LP inoculation modalities, dropping below 10^6^ CFU/mL by Day 5 (Figure 1C). Such a decrease might be attributed to the inhibitory effect of the multi-stressor wine environment, especially ethanol and high wine acidity [16]. Ethanol interacts with bacterial cell membranes, consequently perturbing membrane structure and cell function, whilst high acidity can impair ATPase activity and hexose metabolism in lactic acid bacteria [20]. Of particular interest was the fact that bacterial numbers of PreAF-LP were 2–20-fold higher compared to those of S-LP throughout fermentation (*p* = 0.0327, Students’ *t*-test), indicating the role of inoculation timing on bacterial viability. Differential yeast–bacteria interaction patterns between these two LP inoculation regimes may result in this phenomenon [19], but the corresponding analysis was beyond the scope of the current study.

Compared to the control groups (D-SC and TA-SC), inoculation of LP induced a relatively slower fermentation onset (Figure 1D). Nonetheless, all tested batches fermented to dryness, with less than 4 g/L residual sugar when fermentation terminated. The fastest fermentation was displayed by D-SC (6 days), whilst the rest three batches took one day longer to deplete sugar (Figure 1D). Since very marginal difference was observed in yeast population amongst the four treatments (Figure 1B), delay in alcoholic fermentation in both the bio-acidified and chemical acidified treatments were likely to be the result of less efficient sugar metabolism by yeast compared to the D-SC control. Considering the comparable fermentation duration between LP and TA-SC wines, yeast–bacteria interaction alone is unlikely to reduce the sugar metabolism efficiency of SC in LP treatments, but rather in combination with the inhibitory effect of increased TA [19].

### 3.2. Oenological Parameters of the Cabernet Sauvignon Wines

The impact of LP inoculation prior to/simultaneously with SC on main oenological parameters were examined at the end of the Cabernet Sauvignon fermentations (Table 1). All LP inoculation treatments and SC groups fermented to dryness, with residual sugar ranging between 1.06 (TA-SC) and 2.26 (PreAF-LP) g/L. Further analysis on main organic acids in wines show that the largest variation was found for lactic acid, which were ~5-fold higher in LP wines compared with the D-SC wines (0.4 g/L) (Table 1). In the latter, lactic acid concentration (0.4 g/L) was likely associated with partial degradation of malic acid by autochthonous microbes, which agrees with the stoichiometry of MLF (0.67 g lactic acid is converted from 1 g malic acid) [9], so did the TA-SC treatment (Table 1 and Table S1).

As a result of LP introduction, more degradation of malic acid but incomplete MLF was observed for both LP treatments (Table 1), whilst previous studies reported ultimate utilisation of malic acid by co-inoculations of LP and SC multi-cultures [23,25]. Lactic acid bacteria utilise malic acid to support cell growth [40], and bacterial population, which in turn would exert large influences on the success of MLF. Empirically, viable bacterial numbers less than 10^6^ CFU/mL can pose a potential risk of stuck or protracted MLF. As described above, here, reduced bacterial viability may cause MLF stalled from Day 5 onwards (Figure 1C, Table 1). Taken together, lactic acid in LP wines were from both lactic and MLF, and the former can be related to the time lapse between LP and SC inoculations [14].

Based on calculation using the stoichiometry of MLF, lactic acid produced from hexose metabolism was approximately 1.14 g/L for the S-LP treatment, and 1.31 g/L when SC was inoculated 1 day after LP (Table 1). Greater levels of lactic acid might be yielded if SC was added a few days later. However, as inoculation of LP easily enabled the onset of MLF, both MLF and lactic fermentation should be carefully taken into account when using LP to ameliorate wine acidity [13]. In this study, the acidifying lactic fermentation outcompeted the de-acidifying MLF by LP, leading to the increase in TA in LP wines. The highest TA was observed in PreAF-LP wines (8.04 g/L), followed by TA-SC (7.72 g/L) and S-LP (7.58 g/L), whilst that of the D-SC wines was the lowest (6.62 g/L) (Table 1). Correspondingly, the three latter groups also possessed a slightly lower wine pH compared to D-SC, agree with previous studies [13,14].

Bio-acidification during winemaking had been extensively investigated using the acidifying yeast *L. thermotolerans*, whose lactic acid yield can be up to 16.6 g/L under oenological conditions [11]. Similarly to LP in this study, levels of lactic acid formed by the same *L. thermotolerans* strain can be depended on inoculation regimes with SC. Lesser amounts of lactic acid can be seen in wines fermented by the co-inoculation modality (e.g., 0.6~8.9 g/L, [9]) compared to the sequentially inoculated fermentations (e.g., 1.0~11 g/L, [9]). Such differences in lactic acid yield can be due to the antagonistic activities of SC against *L. thermotolerans*. Compared to *L. thermotolerans* reported in previous studies, the LP strain in this study showed comparable or less lactic acid production, depending partially on the yeast strain being evaluated [9,10,11]. Similarly, lactic acid yield by LP can be strain specific, and further studies on screening of superior LP strains with desired lactic fermentation performance are underway. In addition, for *L. thermotolerans*, factors such as fermentation temperature and the presence of SO_2_ also exerted influences on lactic acid production [10]. Since these physico-chemical factors also affect the growth and metabolism of LP, it is worth investigating the response of LP to these factors upon lactic fermentation.

Relating to the presence of LP, all wines were quantified for volatile acidity, which was between 0.31 and 0.52 among tested wines (Table 1), conforming to the approved National Standard of the People’s Republic of China (GB/T 15038-2006). Volatile acidity was contributed primarily from acetic acid, which was found higher in LP wines compared to SC wines (Table 1). Nonetheless, these amounts are unlikely to be detrimental to wine flavour as they were well below the sensory threshold (0.5 g/L) [41]. In theory, extra acetic acid formation through lactic fermentation by LP can be avoided due to its homofermentative hexose metabolism. Thus, the slight increase in acetic acid in LP wines are likely due to catabolism of other organic acids mediated by the bacteria, possibly through citric acid metabolism (Table 1) [16]. Tartaric acid remained rather stable in all wine samples, with LP inoculation inducing a weak but not significant decrease in this organic acid compared to the D-SC control (*p* > 0.05 in each scenario, Tukey’s HSD post hoc analysis, Table 1).

The control TA-SC yielded the highest concentration of ethanol (12.85% *v*/*v*), comparable to those of D-SC. Notedly, co-inoculation of LP had up to 0.4% *v*/*v* less ethanol than the SC controls, but no statistical difference was detected between these wines. In comparison, a further and significant decrease in ethanol was recorded in the PreAF-LP treatment (up to 1.2% *v*/*v*), coinciding with a study reported by Lucio et al. [13]. Lower ethanol concentration in co-cultured wines was in accord with the partial conversion of sugars to lactic acid by LP, the extent of which varied partially due to inoculation timing (Table 1) and the choice of LP strains [13].

### 3.3. Volatile Composition of the Cabernet Sauvignon Wines

Cabernet Sauvignon wine samples were collected at the end of wine fermentations and were subjected to volatile analysis. A total of 51 volatile compounds were identified and quantified in all wines using HS-SPME-GC-MS, and the concentration is displayed in Table 2 and Table S2. The volatiles detected were further classified into 8 categories, including 4 acetate esters, 15 ethyl esters, 4 other esters, 14 higher alcohols, 5 fatty acids, 2 aldoketones, 1 C13-norispoprenoid, 5 terpenes, and 1 lactone (Table 2). One-way ANOVA and Tukey’s post hoc analysis was used to evaluate statistical differences among all volatiles. All compounds except 1-octen-3-ol showed significant differences among wine samples produced using the four fermentation modalities (Table 2). Besides volatile concentrations, the analytes were also determined for their odour activity values (OAV), which is widely used to indicate the contribution of each compound to wine aroma [9,10,29]. In all wines, concentrations of 14 compounds exceeded their odour thresholds, implying their direct contribution to wine aroma formation. A total of 36 volatiles failed to surpass their detection thresholds, thus imparting sensory perception indirectly via a synergistic effect, which ultimately can lead to global alterations in wine aroma [9,42]. A special scenario was found in isoamyl lactate, which was described as contributing to cream and nut aromas [32]. This compound was detected above its sensory threshold in LP wines, but not the remaining wines produced with SC monocultures (Table 2).

Remarkable differences were found in ester concentrations between wines made by all tested modalities (Table 2). Among the ethyl esters, ethyl acetate and ethyl lactate were the most prevalent in all wines, with both compounds presenting in lower concentrations in SC monoculture wines compared to LP wines. Concentrations of ethyl acetate ranged between 55.398 mg/L (D-SC) and 66.976 (S-LP) mg/L, all were below the level (150 mg/L), at which it was more likely to impart spoilage aromas (i.e., nail polish remover aroma) rather than contributing to fruity notes [33]. A more striking difference was observed for ethyl lactate (fruity and buttery aroma [43]), which was 6 times higher in all LP wines compared to the D-SC wines. Such accumulation can be due to the fact that more lactic acid was available as its precursor in LP wines [9,10]. Ethyl lactate in TA-SC wines also retained at a higher level than those in the D-SC samples, but the increase was to a lesser extent compared to the LP wines (Table 2). Given that lactic acid content in TA-SC was the lowest, formation of the intermediary amount of ethyl lactate may also be related to esterase activities under more acidic conditions [33]. As a result of greater yield of ethyl lactate, the LP wines displayed significantly higher concentrations of total ethyl esters than D-SC and TA-SC wines (Table 2). Nonetheless, concentration of ethyl lactate was detected well below the aroma threshold (146 mg/L [30]) in all wines, indicating that this compound was unlikely to contribute directly to enhance the aromas of LP wines.

Some lesser abundant ethyl esters, e.g., ethyl butanoate, ethyl hexanoate, ethyl octanoate, ethyl isobutyrate, and ethyl isovalerate, were also found to be impact odorants with concentrations surpassing their respective sensory thresholds. These fatty acid ethyl esters were described to contribute to fruity aromas [31], and were generally less in LP wines compared to the SC wines (Table 2). The only exception was observed for ethyl butanoate, which was significantly higher in PreAF-LP wines than SC wines (Table 2). Among the fatty acid ethyl esters, one noticeable effect was a 2–6-fold decrease in ethyl octanoate (pear, apricot aroma [31]) in LP wines than the D-SC wines (Table 2). A further comparison was made between the corresponding fatty acid precursors of the mentioned fatty acid ethyl esters. The LP wines were again notable due to their lower concentrations of octanoic, hexanoic, decanoic, isobutyric and isovaleric acids than those presented in SC wines (Table 2). Contrary to the current study, Devi et al. [22], Tufariello et al. [25], and Brizuela et al. [45] reported several fatty acid ethyl esters and their medium-chain intermediates retained at a higher level in LP/SC mixed culture wines. Together, these findings were in agreement with Saerens et al. [46] that accumulation of fatty acids precursors boosted production of fatty acid ethyl esters. Whilst medium chain fatty acids can hardly be produced by bacteria, these are mainly biosynthesised during yeast lipid metabolism from acetyl-CoA through the fatty acid synthase complex [6]. Of particular interest were the PreAF-LP wines, constantly showing higher concentrations of fatty acids and fatty acid ethyl esters compared to S-LP wines (Table 2). These observations suggest that varied yeast–bacteria interaction patterns resulting from different inoculation regimes may modulate SC upon the release of medium-chain fatty acids available for esterification.

Of the four quantified acetate esters, only isoamyl acetate with banana aroma [30] was found above its sensory perception threshold, and was presented with the highest concentration in D-SC wines followed by TA-SC, PreAF-LP and S-LP wines (Table 2). The D-SC wines were also abundant in isobutyl acetate (banana aroma [31]), hexyl acetate (fruity and floral aroma [30]), and 2-pheylethyl acetate (honey, floral and fruity aroma [31]). As a consequence, the greatest levels of total acetate esters were found in D-SC wines (Table 2). By contrast, the lowest levels of acetate esters were presented in S-LP wines, whilst those in Pre-LP and TA-SC were intermediary (Table 2).

Besides ethyl and acetate esters, four other esters including methyl octanoate, isoamyl hexanoate, 3-methylbutyl octanoate and isoamyl lactate were also identified in Cabernet Sauvignon wines (Table 2). Among these esters, only isoamyl lactate (cream and nut aroma [32]) in LP wines were found exceeding the sensory threshold, and were 9 and 7 times higher in PreAF-LP and S-LP treatments than D-SC, respectively. Again, the trend in isoamyl lactate concentrations were also linked to variation in lactic acid in the tested wines. Another noticeable difference was seen for 3-methylbutyl octanoate (sweet and fruity aroma [30]), which were 2~6 times lower in LP wines compared to the SC wines, albeit present at concentrations below threshold (Table 2).

All four batches of wines contained considerable amounts of higher alcohols, which can potentially mask fruity aromas in red wines [47]. Total concentrations of higher alcohols were comparable between SC and PreAF-LP wines, whilst that of the S-LP wines was much lower (Table 2). Maicas et al. [24] hypothesised that lower concentrations of certain higher alcohols in LP/SC mixed culture wines may be resulted from physical adsorption by the bacteria. Yet, this remained to be verified with the indigenous LP strain used in this study. Among the identified higher alcohols, the most abundant was isobutanol, followed by phenylethyl alcohol and isoamyl alcohol (Table 2). Concentrations of the two former higher alcohols exceeded their sensory thresholds but not isoamyl alcohol (Table 2). The highest level of isobutanol was observed in LP wines, accounting for 80% of their total higher alcohols, whilst TA-SC wines had the least amount and proportion of isobutanol (~66%) (Table 2). Phenylethyl alcohol concentrations were comparable in D-SC and TA-SC wines, which were approximately two times higher than those in LP wines. Another noticeable effect was observed for 1-octanol, whose concentration was the second lowest, but OAVs were markedly greater than any of the higher alcohols (Table 2). The remaining higher alcohols determined in all wines were all below their sensory thresholds, as was the case for aldoketones, terpenes and γ-octalactone. The concentration of β-damascenone in Cabernet Sauvignon wines ranged between 7.75 and 12.61 μg/L, far exceeding its aroma threshold (0.05 μg/L [30]). Notedly, β-damascenone presented in SC wines was approximately 1.3-fold higher than PreAF-LP wines and 1.5-fold higher than S-LP wines (Table 2).

### 3.4. Multivariate Analysis of Cabernet Sauvignon Wine Parameters

**Figure 2:**
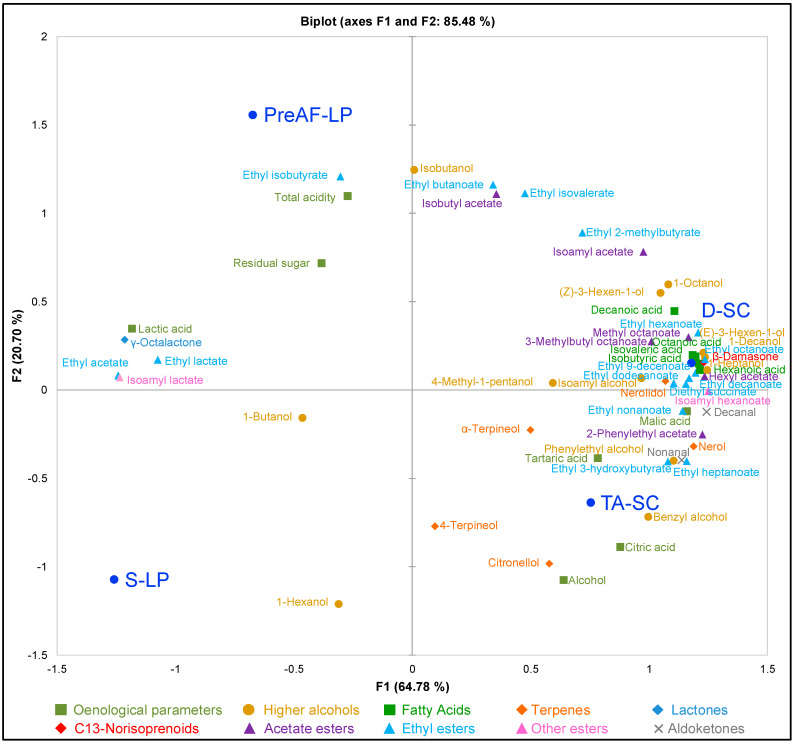
Principal component analysis of oenological parameters and volatile compounds in the Cabernet Sauvignon wines obtained from four different fermentation modalities.

Apart from univariate analysis, i.e., one-way ANOVA and Students’ *t*-test in this study, principal component analysis (PCA) was further conducted to visualise the discrimination of the entire set of chemical data of the resultant wines (Figure 2). The chemical dataset being analysed included both the oenological parameters and the volatile compounds of the Cabernet Sauvignon wines. The first two principal components (PCs) clearly separated the wines fermented using four different vinification modalities and accounted for 85.48% of the overall variation in chemical components (Figure 2). The bio-acidified wines induced by LP inoculations were separated from SC wines on PC1, which explained 64.78% of the variation. Specifically, the LP wines were located at the negative axis of PC1 and were associated with greater concentrations of lactic acid, ethyl lactate, isoamyl lactate, ethyl acetate and γ-octalactone (Figure 2). By contrast, both D-SC and TA-SC were located at the positive axis of PC1 and was associated with a large number of esters, higher alcohols, fatty acids and terpenes (Figure 2). However, a majority of these compounds were presented with concentrations below their sensory thresholds in wines (Table 2). PC2 allows further differentiation between LP treatments, with PreAF-LP located towards the top of the PC2 axis and S-LP towards the bottom (Figure 2). The parameters that appear to be driving the separation of PC2 towards the top were ethyl isobutyrate, isobutanol, ethyl butanoate, isobutyl acetate, ethyl isovalerate and total acidity, whilst 1-hexanol, alcohol, and citronellol drove the separation towards the bottom of the plot (Figure 2). Again, the multivariate analysis demonstrated the ability of LP to induce biological acidification whilst triggering a distinct volatile profile compared to the SC control wines.

### 3.5. Sensory Profiling of the Cabernet Sauvignon Wines

**Figure 3:**
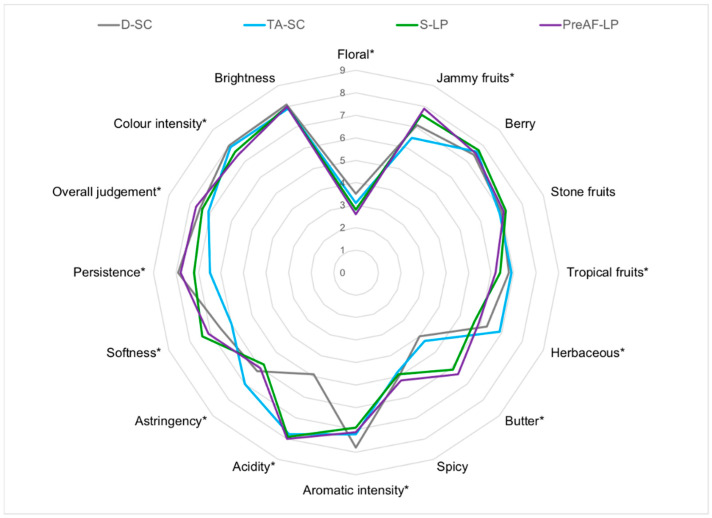
Sensory evaluation of the Cabernet Sauvignon wines obtained from four different fermentation modalities. * indicates perception of certain sensory attributes were significantly different between the four batches of wines. Numbers 0–9 indicates the intensity of each sensory attributes.

A sensory analysis was performed to evaluate all Cabernet Sauvignon wines, and mean scores of the sensory attributes were plotted and shown in Figure 3. The four batches of wines were comparable in brightness; however, they varied in “colour intensity” (Figure 3). Evaluation was further conducted in wine aroma profile, where D-SC wines were found to have the best overall aromatic intensity. In terms of individual aroma traits, no major difference was detected in “berry”, “stone fruits” and “spicy” aromas when comparing the scores between wine samples (Figure 3). Besides those similarities, detectable differences were found in “floral”, “jammy fruits”, “tropical fruits”, “herbaceous” and “butter” aromas. The bio-acidified wines produced with LP were mainly characterised by “jammy fruits” and “butter” notes, whereas the SC wines obtained higher scores in “floral”, “tropical fruits”, and “herbaceous” aromas (Figure 3). The aroma perception of LP wines was in line with their volatile profile (Table 2). In addition, a recent study has shown that co-cultures of yeast and bacteria may trigger a masking effect on fruity aromas [43], whilst Lytra et al. [48] reported the additive effect of lactic traits on jammy fruit aromas but displayed the opposite impact on fresh fruit aromas. All these observations coincided with our findings (Figure 3).

Relatively larger variation was observed in mouthfeel attributes amongst all Cabernet Sauvignon wines. The acidified wines, including LP wines and TA-SC wines scored the highest and comparably in “acidity”, which were up to 1.5 higher than the non-acidified D-SC wines (Figure 3). The TA-SC wines ranked the first in scores for wine “astringency”, followed by D-SC, PreAF-LP and S-LP wines, whilst an opposite trend was found for wine “softness” (Figure 3). Both D-SC wines and PreAF-LP wines had significantly longer mouthfeel persistence compared to S-LP and TA-SC wines (Figure 3). A final judgement was performed on the overall quality of all wines, where PreAF-LP wines were scored the highest (7.7), followed by D-SC (7.5), S-LP (7.4) and TA-SC (7.1) wines (Figure 3).

## 4. Conclusions

Inadequate acidity in grape berries due to climate warming is of increasing concerns for winemaking, and homofermentative LP shows potential to address this issue. This work describes comprehensive characterisation of using an indigenous LP strain to induce biological acidification with two inoculation regimes at pilot-scale vinification. Both LP inoculation strategies yielded considerable amount of lactic acid, which in turn led to increased titratable acidity in Cabernet Sauvignon wines. Further, the addition of LP prior to or simultaneously with SC had little to no impact on the completion of alcoholic fermentation as well as fermentation duration compared to those of chemical acidification treatment. Beyond bio-acidification, LP induced multiple effects on wine volatile profile, in particular the remarkable increase in lactate-related esters. In line with the changes in wine composition, the sensory profile of the bio-acidified wines was shifted towards “jammy fruit” and “butter” notes with better mouthfeel benefited from improved wine acidity. Considering the overall impact on wine fermentation and flavour profile, reverse inoculation of LP was more effective compared to co-inoculation of LP. Although LP enabled enhanced wine acidity via lactic fermentation, the fact that LP also easily enabled MLF should be taken into account when using this tool to induce bio-acidification. Overall effects on wine acidity resulting from combined lactic fermentation and MLF by LP depend on several factors, including inoculation timing. These findings invite further research on thoroughly investigating how the time lapse between LP and SC inoculations influence yeast–bacteria interactions, which ultimately modulates wine acidification and flavour.

## Figures and Tables

**Table 1:** Basic oenological parameters of the Cabernet Sauvignon wines obtained from four different fermentation modalities.

Parameters	D-SC	TA-SC	S-LP	PreAF-LP
Sugar (g/L)	2.19 ± 0.11 b	1.06 ± 0.23 a	1.94 ± 0.17 b	2.26 ± 0.07 b
Lactic acid (g/L)	0.40 ± 0.13 a	0.23 ± 0.13 a	2.04 ± 0.01 b	2.05 ± 0.03 b
Malic acid (g/L)	1.53 ± 0.13 c	1.78 ± 0.04 d	0.81 ± 0.03 a	1.04 ± 0.05 b
Citric acid (g/L)	0.31 ± 0.03 b	0.33 ± 0.03 b	0.27 ± 0.01 b	0.20 ± 0.01 a
Tartaric acid (g/L)	2.93 ± 0.06 a	4.06 ± 0.04 b	2.79 ± 0.12 a	2.83 ± 0.01 a
Acetic acid (g/L)	0.23 ± 0.01 a	0.26 ± 0.01 a	0.37 ± 0.01 ab	0.50 ± 0.01 b
^A^Titratable acidity (g/L)	6.62 ± 0.19 a	7.72 ± 0.06 b	7.58 ± 0.13 b	8.04 ± 0.05 c
pH	3.58 ± 0.01 c	3.55 ± 0.01 b	3.57 ± 0.01 bc	3.51 ± 0.01 a
^B^Volatile acidity (g/L)	0.31 ± 0.03 a	0.32 ± 0.07 a	0.40 ± 0.01 a	0.54 ± 0.08 a
Ethanol (% *v*/*v*)	12.65 ± 0.21 b	12.85 ± 0.21 b	12.55 ± 0.07 b	11.60 ± 0.14 a

Values (mean ± standard deviation) within each row followed by different letters are significantly different according to one-way ANOVA, Tukey’s honest HSD post hoc tests at 95% confidence level. ^A^Titratable acidity is expressed as g/L tartaric acid. ^B^Volatile acidity is expressed as g/L acetic acid.

**Table 2:** Volatile compounds of the Cabernet Sauvignon wines obtained from four different fermentation modalities (µg/L).

Compounds	Aroma Threshold	D-SC		TA-SC		S-LP		PreAF-LP		Odour Description
		Concentration	OAV	Concentration	OAV	Concentration	OAV	Concentration	OAV	
Isoamyl acetate	30 [30]	2277.34 ± 23.14 c	75.9	1833.62 ± 95.75 b	61.1	1020.43 ± 34.48 a	34.0	1976.14 ± 87.67 b	65.9	Banana [30]
Isobutyl acetate	1600 [31]	185.84 ± 5.42 b	0.1	108.29 ± 1.34 a	<0.1	97.80 ± 5.82 a	<0.1	191.18 ± 8.17 b	0.1	Banana [31]
Hexyl acetate	670 [30]	11.86 ± 0.16 c	<0.1	7.82 ± 0.74 b	<0.1	4.25 ± 0.31 a	<0.1	5.37 ± 0.22 a	<0.1	Fruity, floral [30]
2-Phenylethyl acetate	250 [31]	31.31 ± 3.67 b	0.1	29.32 ± 1.39 b	0.1	11.92 ± 0.35 a	<0.1	12.44 ± 0.46 a	<0.1	Honey, floral, fruity [31]
**Σ Acetate esters**		**2506.35 ± 32.39 d**		**1979.05 ± 99.22 b**		**1134.40 ± 40.96 a**		**2185.13 ± 96.52 c**		
Ethyl acetate	7500 [31]	55,398.05 ± 609.17 a	7.4	55,799.40 ± 382.54 a	7.4	66,976.00 ± 958.55 b	8.9	64,385.25 ± 4396.44 b	8.6	Fruity, nail polish, balsamic [33]
Ethyl butanoate	20 [30]	106.29 ± 3.73 b	5.3	103.35 ± 1.23 b	5.2	77.64 ± 4.68 a	3.9	127.72 ± 7.28 c	6.4	Strawberry, lactic [30]
Ethyl hexanoate	14 [30]	903.99 ± 43.24 c	64.6	821.06 ± 51.74 c	58.6	477.62 ± 37.73 a	34.1	684.05 ± 26.34 b	48.9	Apple peel, fruit [30]
Ethyl heptanoate	220 [31]	6.07 ± 0.30 c	<0.1	4.91 ± 0.64 b	<0.1	3.32 ± 0.30 a	<0.1	2.6 ± 0.15 a	<0.1	Fruity, pineapple [31]
Ethyl octanoate	5 [31]	862.82 ± 71.22 d	172.6	546.01 ± 88.56 c	109.2	146.91 ± 1.16 a	29.4	342.8 ± 17.11 b	68.6	Pear, apricot [31]
Ethyl nonanoate	1200 [30]	4.69 ± 0.08 c	<0.1	4.03 ± 0.15 b	<0.1	3.54 ± 0.03 a	<0.1	3.51 ± 0.06 a	<0.1	Waxy, fruity, rose, rum [30]
Ethyl decanoate	200 [31]	130.85 ± 15.01 c	0.7	99.81 ± 14.89 b	0.5	38.26 ± 0.14 a	0.2	59.99 ± 0.21 a	0.3	Fruity, fatty [31]
Ethyl 3-hydroxybutyrate	20,000 [33]	514.45 ± 27.25 b	<0.1	583.44 ± 7.35 c	<0.1	385.1 ± 20.67 a	<0.1	383.11 ± 1.38 a	<0.1	Grape
Ethyl lactate	146,000 [30]	22,375.3 ± 335.03 a	0.2	86,150.35 ± 5320.34 b	0.6	114,821.5 ± 737.51 c	0.8	126,198.7 ± 19.66 c	0.9	Fruity, buttery [43]
Ethyl dodecanoate	500 [30]	101.79 ± 3.13 c	0.2	90.36 ± 1.01 b	0.2	84.28 ± 0.08 a	0.2	85.59 ± 0.83 a	0.2	Sweet, floral, fruity, buttery [30]
Diethyl succinate	1,250,000 [30]	633.88 ± 92.01 bc	<0.1	691.51 ± 16.79 c	<0.1	444.41 ± 2.62 ab	<0.1	533.68 ± 27.01 a	<0.1	Wine, fruity [30]
Ethyl isobutyrate	15 [34]	206.43 ± 3.06 b	13.8	160.38 ± 1.68 a	10.7	173.24 ± 11.77 a	11.5	288.74 ± 14.44 c	19.2	Fruity, strawberry, lemon [33]
Ethyl 2-methylbutyrate	18 [31]	28.82 ± 0.97 b	1.6	30.46 ± 0.94 b	1.7	17.54 ± 1.72 a	1.0	32.20 ± 2.16 b	1.8	Apple, berry, sweet, cider, anise [33]
Ethyl isovalerate	3 [31]	37.55 ± 6.58 b	12.5	35.64 ± 0.56 b	11.9	18.08 ± 1.66 a	6.0	47.72 ± 1.85 c	15.9	Banana, sweet, fruity [33]
Ethyl 9-decenoate	100 [36]	40.54 ± 1.71 c	0.4	34.07 ± 1.5 b	0.3	26.6 ± 0.11 a	0.3	29.34 ± 0.08 a	0.3	Fruity, fatty [30]
**Σ Ethyl esters**		**81,351.52 ± 1212.49 a**		**145,154.78 ± 5889.92 b**		**183,694.04 ± 1778.73 c**		**193,204.95 ± 4515.00 d**		
Methyl octanoate	4 [30]	4.04 ± 0.19 c	1.0	2.72 ± 0.34 b	0.7	1.26 ± 0.03 a	0.3	2.20 ± 0.06 b	0.6	Orange [30]
Isoamyl hexanoate	NF	6.40 ± 0.21 b	ND	6.24± 0.28 b	ND	5.17 ± 0.03 a	ND	5.48 ± 0.05 a	ND	Apple, green, pineapple
3-Methylbutyl octanoate	125 [30]	12.06 ± 2.14 c	<0.1	8.01 ± 0.07 b	<0.1	2.25 ± 0.11 a	<0.1	4.77 ± 0.06 a	<0.1	Sweet, fruity, pineapple, coconut [30]
Isoamyl lactate	200 [32]	42.07 ± 2.1 a	0.2	36.93 ± 0.12 a	0.2	370.22 ± 8.48 c	1.9	287.17 ± 2.98 b	1.4	Cream, nut [32]
**Σ Other esters**		**64.57 ± 24.64 a**		**53.90 ± 0.81 a**		**378.90 ± 8.65 c**		**299.62 ± 3.15 b**		
1-Hexanol	8000 [30]	1786.34 ± 5.66 b	0.2	1979.48 ± 99.07 c	0.2	2214.96 ± 3.90 d	0.3	1558.28 ± 13.77 a	0.2	Green, grass [30]
(E)-3-Hexen-1-ol	1000 [35]	122.83 ± 5.88 b	0.1	146.33 ± 12.54 c	0.1	77.65 ± 4.92 a	0.1	115.23 ± 0.82 b	0.1	Herbaceous, green [35]
(Z)-3-Hexen-1-ol	400 [37]	272.50 ± 2.62 c	0.7	213.96 ± 15.38 b	0.5	167.49 ± 4.00 a	0.4	218.19 ± 1.80 b	0.5	Green, cypress [37]
1-Butanol	150,000 [31]	1437.36 ± 9.67 a	<0.1	2034.81 ± 38.34 c	<0.1	1819.35 ± 41.05 b	<0.1	1873.73 ± 78.44 b	<0.1	Fusel alcohol [31]
Isobutanol	40,000 [31]	240,292 ± 8763.88 b	6.0	219,126 ± 371.94 a	5.5	214,157 ± 6051.42 a	5.4	264,662.50 ± 3293.00 c	6.6	Fusel alcohol [31]
Isoamyl alcohol	30,000 [31]	25,219.50 ± 1914.14 b	0.8	27,527.20 ± 12,880.66 c	0.9	22,637.00 ± 3289.46 a	0.8	24,621.95 ± 4541.75 b	0.8	Whisky, nail polish [31]
4-Methyl-1-pentanol	50,000 [37]	66.66 ± 3.31 a	<0.1	89.83 ± 3.77 c	<0.1	60.78 ± 0.28 ab	<0.1	72.97 ± 0.43 b	<0.1	Almond, toasted [37]
3-Methyl-1-pentanol	500 [31]	167.26 ± 0.09 a	0.3	255.75 ± 9.50 b	0.5	168.49 ± 4.16 a	0.3	174.85 ± 0.91 a	0.3	Soil, mushroom [31]
1-Heptanol	200–300 [31]	58.16 ± 1.05 c	0.1–1	50.52 ± 2.86 b	0.1–1	39.37 ± 1.07 a	0.1–1	43.73 ± 0.85 a	0.1–1	Lemon, orange, copper [31]
1-Octanol	0.7 [30]	18.03 ± 0.42 c	25.8	16.27 ± 1.46 bc	23.2	3.81 ± 0.14 a	5.4	14.29 ± 0.67 b	20.4	Chemical, metal, burnt [30]
1-Octen-3-ol	40 [39]	4.59 ± 0.05 a	0.1	6.09 ± 2.38 a	0.2	4.42 ± 0.21 a	0.1	4.25 ± 1.35 a	0.1	Mushroom [39]
1-Decanol	500 [30]	2.74 ± 0.33 a	<0.1	2.45 ± 0.19 b	<0.1	1.56 ± 0.02 a	<0.1	1.98 ± 0.02 ab	<0.1	Fat [30]
Benzyl alcohol	200,000 [31]	158.81 ± 18.79 bc	<0.1	174.62 ± 15.64 c	<0.1	131.11 ± 2.5 ab	<0.1	109.08 ± 5.18 a	<0.1	Almond [31]
Phenylethyl alcohol	14,000 [31]	62,278.00 ± 1426.38 b	4.4	78,121.80 ± 2987.81 c	5.6	38,050.10 ± 157.68 a	2.7	37,875.55 ± 2014.48 a	2.7	Floral, rose [31]
**Σ Higher alcohols**		**331,884.78 ± 12,152.27 b**		**329,745.11 ± 16,441.54 b**		**279,533.09 ± 9560.81 a**		**331,346.58 ± 7944.17 b**		
Octanoic acid	500 [30]	2282.31 ± 635.02 c	4.6	2048.17 ± 181.76 bc	4.1	502.56 ± 3.08 a	1.0	1263.75 ± 151.54 ab	2.5	Butter, almond [30]
Decanoic acid	1000 [30]	50.41 ± 2.82 b	<0.1	51.78 ± 1.74 b	<0.1	28.20 ± 0.49 a	<0.1	44.25 ± 7.08 b	<0.1	Rancid, fat [30]
Hexanoic acid	420 [30]	2877.46 ± 493.75 b	6.9	2482.3 ± 246.27 b	5.9	1121.08 ± 13.41 a	2.7	1663.79 ± 116.52 a	4.0	Leafy, wood, varnish [30]
Isobutyric acid	200,000 [37]	6796.09 ± 949.58 c	<0.1	5712.2 ± 335.31 bc	<0.1	3447.21 ± 149.45 a	<0.1	4449.45 ± 281.37 ab	<0.1	Cheese, butter, rancid [37]
Isovaleric acid	33.4 [37]	18.31 ± 1.33 c	0.5	19.52 ± 0.71 c	0.6	6.20 ± 0.00 a	0.2	12.60 ± 0.24 b	0.4	Fatty, sweet [37]
**Σ Fatty acids**		**12,024.58 ± 2082.50 c**		**10,313.97 ± 765.79 bc**		**5105.25 ± 166.43 a**		**7433.84 ± 556.75 ab**		
Nonanal	2.5 [30]	0.71 ± 0.06 b	0.3	0.54 ± 0.06 b	0.2	0.20 ± 0.01 a	<0.1	0.08 ± 0.21 a	<0.1	Fat, citrus, green [30]
Decanal	1.25 [38]	0.93 ± 0.10 b	0.7	0.80 ± 0.08 b	0.6	0.32 ± 0.05 a	0.3	0.39 ± 0.11 a	0.3	Green [38]
**Σ Aldoketones**		**1.64 ± 0.16 b**		**1.34 ± 0.14 b**		**0.52 ± 0.06 a**		**0.47 ± 0.32 a**		
β-Damascenone	0.05 [30]	12.61 ± 0.68 d	252.2	11.29 ± 0.37 c	225.8	7.75 ± 0.09 a	155.0	9.38 ± 0.17 b	187.6	Apple, rose, honey [30]
**Σ C13-Norisoprenoids**		**12.61 ± 0.68 d**		**11.29 ± 0.37 c**		**7.75 ± 0.09 a**		**9.38 ± 0.17 b**		
α-Terpineol	250 [30]	20.11 ± 2.53 c	<0.1	1.05 ± 0.10 a	<0.1	9.00 ± 0.10 b	<0.1	1.04 ± 0.22 a	<0.1	Oil, anise, spicy [30]
4-Terpineol	250 [37]	2.11 ± 0.05 b	<0.1	1.13 ± 0.33 a	<0.1	2.10 ± 0.05 b	<0.1	0.76 ± 0.04 a	<0.1	Flowery [37]
Citronellol	100 [30]	2.76 ± 2.41 ab	<0.1	5.39 ± 0.17 b	<0.1	3.00 ± 0.07 ab	<0.1	0.53 ± 0.24 a	<0.1	Citrus [30]
Nerol	500 [32]	3.90 ± 0.25 c	<0.1	3.44 ± 0.10 b	<0.1	2.48 ± 0.01 a	<0.1	2.30 ± 0.03 a	<0.1	Violets, floral [32]
Nerolidol	700 [37]	3.29 ± 0.15 c	<0.1	2.49± 0.03 b	<0.1	2.16 ± 0.00 a	<0.1	2.23 ± 0.03 a	<0.1	Rose, apple, citrus [37]
**Σ Terpenes**		**32.17 ± 5.39 c**		**13.5 ± 0.73 ab**		**18.74 ± 0.23 b**		**6.86 ± 0.56 a**		
γ-Octalactone	400 [34]	26.02 ± 0.03 a	<0.1	26.01 ± 0.01 a	<0.1	46.72 ± 0.24 b	0.1	46.22 ± 1.68 b	0.1	Coconut [44]
**Σ Lactones**		**26.02 ± 0.03 a**		**26.01 ± 0.01 a**		**46.72 ± 0.24 b**		**46.22 ± 1.68 b**		

Values (mean ± standard deviation) within each row followed by different letters are significantly different according to one-way ANOVA, Tukey’s honest HSD post hoc tests at 95% confidence level. NF, not found. ND, not determined. Boldfaced items are the sum of volatile compounds identified and quantified in each category.

## Data Availability

Data is contained within the article or Supplementary Materials.

## Supplementary Materials

The following supporting information can be downloaded at: https://www.mdpi.com/article/10.3390/foods11162511/s1, Table S1: Physico-chemical parameters of the Cabernet Sauvignon grape must. Table S2: Qualitative information about the analysed volatile compounds in Cabernet Sauvignon wines.
