# Supplementary material for: Pilot-Scale Vinification of Cabernet Sauvignon Using Combined Lactiplantibacillus plantarum and Saccharomyces cerevisiae to Achieve Wine Acidification
Source: Foods. 2022 Aug 19;11(16):2511. doi: 10.3390/foods11162511 (PMC9407048; doi:10.3390/foods11162511)
Supplement: Supplementary file 1 [file foods-11-02511-s001.zip › Table S1 .pdf]

Table S1 Physio-chemical parameters of the Cabernet Sauvignon grape must

Parameters	Cabernet Sauvignon must
Sugar (g/L)	250.00 ± 0.00
Ethanol (% v/v)	ND
[A]Total acidity (g/L)	5.10 ± 0.00
pH	3.70 ± 0.01
[B]Volatile acidity (g/L)	ND
Acetic acid (g/L)	ND
Lactic acid (g/L)	ND
Malic acid (g/L)	2.15 ± 0.01
Tartaric acid (g/L)	3.05 ± 0.01
Citric acid (g/L)	0.28 ± 0.02

ND, not detected; [A]Total acidity is expressed as g/L tartaric acid; [B]Volatile acidity is expressed as g/L acetic acid.
